# Software-based concordance analysis of a predictive surgical planning model for target gap achievement in robotic-assisted total knee arthroplasty

**DOI:** 10.1186/s43019-026-00357-x

**Published:** 2026-11-05

**Authors:** Mingwei Hu, Muyang Yu, Fanyu Meng, Ziyuan Luo, Yuanqing Xia, Xisheng Weng, Dihua Zhai, Bin Feng

**Affiliations:** 1 Department of Orthopedics, Peking Union Medical College Hospital, Chinese Academy of Medical Sciences and Peking Union Medical College, Beijing, China; 2 The School of Automation, Beijing Institute of Technology, Beijing, China; 3 Zhongyuan University of Technology, Zhengzhou, China; 4 State Key Laboratory of Complex Severe and Rare Diseases, Peking Union Medical College Hospital, Chinese Academy of Medical Sciences and Peking Union Medical College, Beijing, China

**Keywords:** Robotic-assisted surgery, Total knee arthroplasty, Predictive modeling, Decision support systems, Surgical planning, Gap balancing

## Abstract

**Background:**

In robotic-assisted total knee arthroplasty (TKA), achieving soft-tissue balance requires repeated intraoperative plan adjustment and remains experience-dependent. Predictive models may support planning, but their clinical value depends on whether model-generated plans approximate predefined target gaps within the robotic workflow. This study assessed the software-based concordance between model-generated plans and predefined target gaps in robotic-assisted TKA. The predefined target gaps were planning set points established within the robotic software rather than measured intraoperative gaps.

**Methods:**

This retrospective software-based concordance study included 209 complete-case knees undergoing robotic-assisted TKA. A machine-learning-based predictive model generated a bone-resection plan, which was imported into the robotic planning software to calculate software-derived medial and lateral gaps in extension and flexion. These software-derived gaps were compared with predefined target gaps, which were planning set points rather than measured values and were 18 mm in 819 of 836 gap-level observations (98.0%). The primary analysis focused on target gap deviation, defined as the software-derived gap minus the predefined target gap. As a benchmark, the deviation of the preadjustment gaps from the same targets was used as a preadjustment reference.

**Results:**

A total of 836 gap-level observations were analyzed. The overall mean target gap deviation was −0.142 mm (SD, 1.411 mm), with a mean absolute error of 1.016 mm. Overall, 77.6% of software-derived gaps were within ±1 mm of target, and 93.4% were within ±2 mm. Bland–Altman analysis showed a small overall bias of −0.142 mm, with 95% limits of agreement from −2.91 to +2.62 mm. At the knee level, only 43.1% of knees had all software-derived gaps within ±1 mm of target. Gap-level deviations ranged from −7 mm to +5 mm, and 16.3% of knees (34/209) had at least one gap with an absolute deviation > 2 mm. Compared with the preadjustment reference (MAE 2.002 mm, 49.4% within ±1 mm), the model substantially reduced deviation (MAE 1.016 mm, 77.6% within ±1 mm, *p* < 0.001).

**Conclusions:**

In this software-based concordance analysis, the predictive planning model generated surgical plans whose robotic software-derived gaps approximated predefined target gaps with a mean absolute deviation of approximately 1 mm. The model-generated plan reduced target-gap deviation relative to the initial preadjustment plan within this target-conditioned workflow. However, because the targets were near-uniform (98.0% at 18 mm) and all measurements were software-derived, compartment-specific bias, moderate whole-knee consistency (43.1%), and occasional outlier deviations indicate that the model should be used as a surgeon-supervised decision support tool rather than as an autonomous planner. Prospective validation against measured intraoperative and postoperative gaps is required before clinical effectiveness can be claimed.

**Supplementary Information:**

The online version contains supplementary material available at https://doi.org/10.1186/s43019-026-00357-x.

## Introduction

Total knee arthroplasty (TKA) is an effective procedure for end-stage knee osteoarthritis and reliably improves pain and function. Achieving appropriate soft-tissue balance remains a central challenge in TKA. To obtain a stable and functional knee, surgeons must integrate multiple variables, including femoral and tibial resection depth, posterior slope, component rotation, and implant sizing, while anticipating their combined effects on medial and lateral gaps in both flexion and extension. In practice, this often requires iterative plan adjustment and can be cognitively demanding, particularly for less experienced surgeons. Robotic-assisted TKA (raTKA) has improved the precision and reproducibility of surgical execution. Recent systematic reviews, meta-analyses, and randomized or comparative studies consistently report superior radiographic or component-positioning accuracy with robotic-assisted TKA compared with conventional techniques, although improvements in short-term clinical outcomes are generally smaller and less consistent [1–8].

Nevertheless, a substantial proportion of patients remain dissatisfied after surgery, and factors such as inaccurate component positioning, suboptimal alignment, and inadequate soft-tissue balancing continue to contribute to less favorable outcomes [9, 10]. Even with more accurate execution, formulation of the intraoperative surgical plan remains complex. Robotic systems make this process more quantitative, but they do not eliminate the need for surgical judgment. Predictive modeling may help address this challenge by providing intraoperative decision support. Recent AI-focused reviews also highlight the promise of machine learning for surgical planning, implant sizing, and individualized decision support while emphasizing the need for rigorous validation in clinically meaningful workflows [11–13]. A model trained on prior surgical data may learn associations between surgical planning variables and resulting soft-tissue gap behavior. In principle, such a model could provide a data-driven starting point for plan refinement and reduce repeated trial-and-error adjustment, although whether it reproduces truly individualized, patient-specific balancing requires testing across a range of target strategies rather than a single near-uniform target. However, the usefulness of this type of model should not be judged solely by whether its generated resection parameters resemble the surgeon’s final choices. Its clinical value depends more directly on whether the model-generated plan, once returned to the robotic system, produces software-derived gaps that correspond to the predefined target gaps. In this context, the target gap is a predefined planning set point established within the robotic workflow, not a measured intraoperative value.

Accordingly, this study was designed as a software-based concordance analysis of target gap achievement rather than as a conventional parameter-prediction analysis. The primary objective was to quantify the concordance between the software-derived gaps produced by the model-generated plan and predefined target gaps within a robotic planning feedback workflow. To place this concordance in context, the preadjustment gaps were used as a preadjustment benchmark.

## Methods

### Study design and setting

This retrospective software-based concordance study was conducted at a single high-volume academic orthopedic center. The study was approved by the institutional review board of the participating center (approval number blinded for peer review). All procedures were conducted in accordance with institutional ethical standards and the Declaration of Helsinki.

### Analytical cohort

We retrospectively screened 237 consecutive TKA procedures performed between September 2024 and March 2026. Patients undergoing primary TKA using a commercially available robotic-arm-assisted system (MAKO, Stryker, Mahwah, NJ, USA) were eligible for inclusion. Of the 237 screened procedures, 2 revision TKAs were excluded, 23 were excluded because of severe deformity that precluded completion of the standard robotic-assisted surgical workflow, and 3 were excluded because of incomplete target-gap data. Severe deformity was defined as cases in which the standard registration, gap-capture, or planning steps could not be completed, such as a fixed flexion contracture greater than 20° that could not be corrected after soft-tissue release and therefore prevented acquisition of a valid gap display, or marked bone loss. The final complete-case analytic cohort therefore comprised 209 knees. Each knee contributed four gap-level observations—medial extension, lateral extension, medial flexion, and lateral flexion, yielding 836 observations for the primary analysis.

### Predictive model

The study evaluated a proprietary machine-learning-based predictive model developed to generate case-specific resection and component-orientation parameters for raTKA. The model was trained on a separate retrospective dataset of 375 primary raTKA cases performed at the same institution between November 2023 and September 2024. Although the training and analytic cohorts did not overlap at the case level, they shared the same center and surgeons; therefore, the present study represents internal concordance testing rather than independent external validation. The training cases incorporated personalized alignment philosophies, including functional alignment and restricted kinematic alignment [14–16].

At inference, the model used 17 variables, comprising 13 preadjustment planning variables and 4 predefined target-gap variables. The preadjustment variables included initial resection and component-orientation parameters and preadjustment medial and lateral gap measurements in extension and flexion. The model generated recommended resection and component-orientation parameters, which were returned to the robotic planning software for calculation of the corresponding software-derived gaps. Variable definitions and measurement sources are provided in Supplementary Table S1. Importantly, the four predefined target gaps served both as model inputs and as the reference for concordance assessment. Accordingly, the present analysis evaluates target-conditioned software-based concordance rather than target-independent generalization. Preadjustment and surgeon-approved final plans were used only as contextual benchmarks.

### Planning feedback workflow

**Fig. 1:**
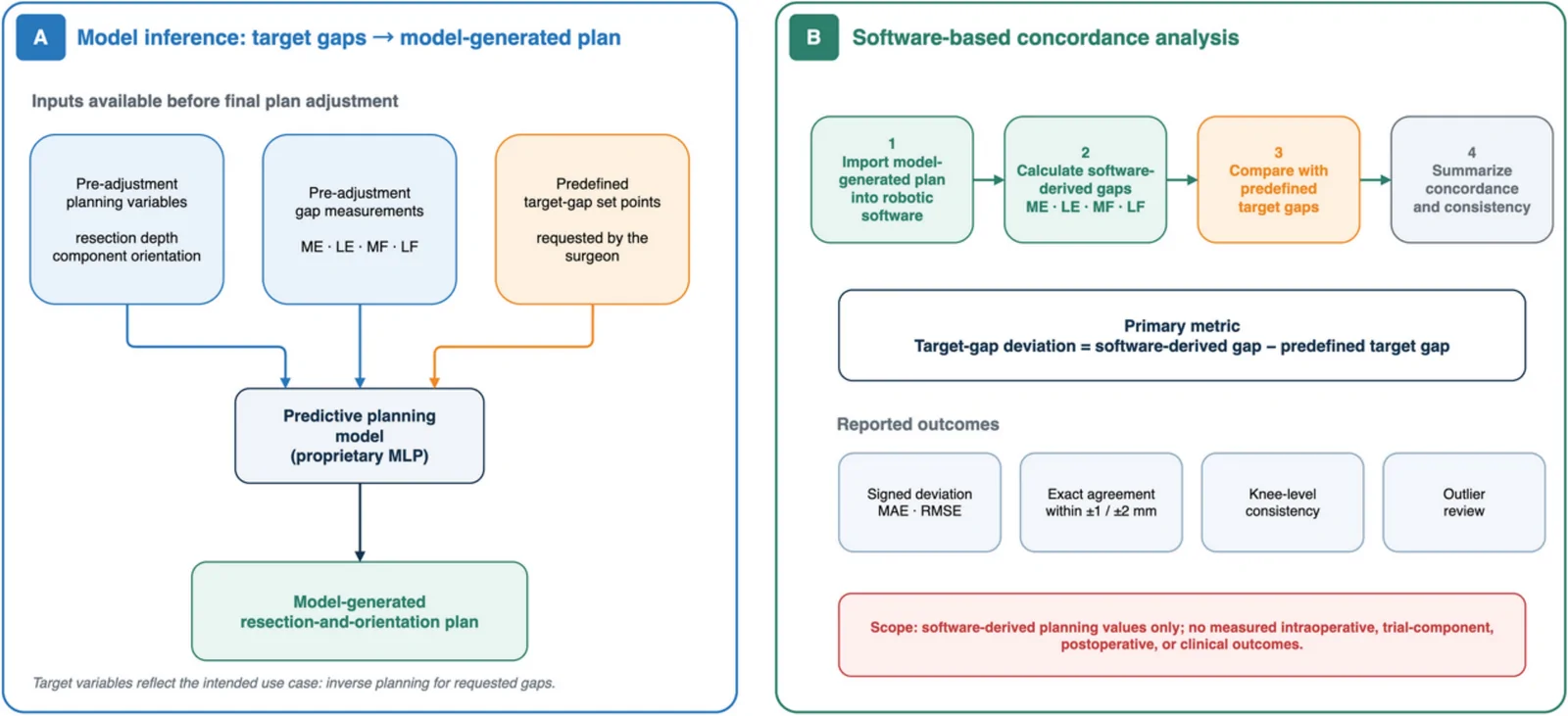
Overview of the predictive planning model and the software-based concordance workflow. In panel **A**, blue indicates preadjustment planning inputs, orange indicates predefined target-gap set points, gray indicates the predictive model, and green indicates the model-generated plan. In panel **B**, green indicates software-processing steps, orange indicates comparison with predefined target gaps, gray indicates concordance summarization, and red indicates the scope limitation of the analysis. Arrows indicate workflow direction. ME, medial extension; LE, lateral extension; MF, medial flexion; LF, lateral flexion

The concordance workflow was designed to assess the model within the robotic planning-feedback loop. For each case, predefined target gaps were established within the robotic workflow. In the present cohort, these targets were 18 mm in 819 of 836 gap-level observations (98.0%) and were not differentiated by compartment (18 mm in 202/209 medial extension, 201/209 lateral extension, 208/209 medial flexion, and 208/209 lateral flexion targets). The 18-mm target reflects our institution’s standard practice, in which the preresection gap is set according to the combined thickness of the femoral component and tibial baseplate-plus-insert construct, consistent with the system manufacturer’s guidance. Deviation patterns across compartments were therefore interpreted in the context of this nearly uniform target setting. The predictive model then generated a complete bone-resection plan based on patient-specific input data. This model-generated plan was programmatically fed back into the robotic planning software, which calculated the virtual medial and lateral gaps in extension and flexion expected from that plan. These software-derived gaps formed the basis of the present evaluation and were compared directly with the predefined target gaps. A schematic overview of the predictive planning model and the software-based concordance workflow is shown in Fig. 1.

### Outcome definitions

The primary outcome was target gap deviation, defined for each compartment as:

Target gap deviation (mm) = software-derived gap from the model-generated plan (mm) − predefined target gap (mm).

A negative deviation indicated a software-derived gap that was smaller than the target, whereas a positive deviation indicated a larger-than-target gap. Signed and absolute deviations were evaluated separately for medial extension, lateral extension, medial flexion, and lateral flexion. Threshold-based target-gap achievement was defined as a software-derived gap within ±1.0 mm or ±2.0 mm of the predefined target. Because the robotic software records gaps in 1-mm increments, exact agreement was defined as a 0-mm deviation and was reported descriptively. The ±1.0-mm threshold was prespecified as the primary pragmatic tolerance, with ±2.0 mm used as a more permissive sensitivity threshold.

Knee-level outcomes included per-knee MAE, the proportion of knees with all four software-derived gaps within ±1.0 mm or ±2.0 mm of target, and the proportions with at least three or at least two gaps within ±1.0 mm. Cases with an absolute target-gap deviation > 2 mm in any compartment were reviewed descriptively as outliers.

### Statistical analysis

All statistical analyses were performed in R version 4.5.3 (R Foundation for Statistical Computing, Vienna, Austria) using lme4, lmerTest, tidyverse, and ggplot2. Two-sided *p* values < 0.05 were considered statistically significant.

Model performance was summarized overall and by gap type using signed target gap deviation, absolute deviation, MAE, RMSE, median absolute error, and the proportions of software-derived gaps within ±1.0 mm and ±2.0 mm of target. Exact agreement was defined as a 0-mm deviation and reported descriptively. Wilson confidence intervals were calculated for threshold-based proportions. Bland–Altman analysis was used to summarize bias and 95% limits of agreement. Correlation-based metrics were not used as primary performance measures given the near-uniform target gap distribution.

Mixed-effects models with knee as a random intercept were used to account for the four gap measurements within each knee. Linear mixed-effects models assessed signed and absolute target gap deviation, and mixed-effects logistic regression assessed within ±1.0-mm target gap achievement. Exploratory descriptive subgroup analyses examined patient sex, target gap category (18 mm versus non-18 mm), and preoperative coronal deformity magnitude (≤ 5° versus > 5°). These analyses were used to characterize performance across strata and were not intended for formal between-group inference.

To contextualize model performance, two reference comparisons were performed. First, preadjustment gaps and model-generated software-derived gaps were compared against the same predefined targets, with paired absolute errors compared using the Wilcoxon signed-rank test. Second, model-generated software-derived gaps were compared with surgeon-approved final software-derived gaps. This final plan reference analysis summarized signed and absolute model-to-final differences, threshold agreement, target gap achievement before and after final plan adjustment, and outlier correction. It was not considered validation against measured intraoperative gaps or postoperative outcomes.

Model-generated resection depths were reviewed as a feasibility screen. Individual bony resections > 10 mm and negative resection values were flagged for review. This screen was limited to resection depth and did not comprehensively assess alignment, tibial slope, femoral flexion–extension, or joint-line consequences.

## Results

### Analytical cohort and preadjustment benchmark

The final complete-case analytic cohort comprised 209 knees, yielding 836 gap-level observations across medial extension, lateral extension, medial flexion, and lateral flexion. Target gaps were 18 mm in 819 of 836 gap-level observations (98.0%). Baseline demographic, deformity, implant, and target gap characteristics are summarized in Table 1.

**Table 1:** Baseline characteristics of the analytic cohort

Characteristic	Value
Cohort size	
Patients, *n*	190
Knees analyzed, *n*	209
Gap-level observations, *n*	836
Demographics	
Female sex, *n*/*N* (%)	147/209 (70.3%)
Male sex, *n*/*N* (%)	62/209 (29.7%)
Age, years, mean ± SD (range)	65.3 ± 10.7 (22 to 88)
BMI, kg/m^2^, mean ± SD (range)	26.3 ± 3.6 (17.0 to 37.8)
ASA physical status	
Class I, *n*/*N* (%)	1/209 (0.5%)
Class II, *n*/*N* (%)	169/209 (80.9%)
Class III, *n*/*N* (%)	39/209 (18.7%)
Clinical characteristics	
Diagnosis: osteoarthritis, *n*/*N* (%)	177/209 (84.7%)
Diagnosis: rheumatoid arthritis, *n*/*N* (%)	13/209 (6.2%)
Diagnosis: other, *n*/*N* (%)	19/209 (9.1%)
Left knee, *n*/*N* (%)	96/209 (45.9%)
Right knee, *n*/*N* (%)	113/209 (54.1%)
Preoperative range of motion, degrees, mean ± SD (range)	102.4 ± 16.9 (20 to 150)
Preoperative alignment characteristics	
Signed coronal deformity, degrees, mean ± SD (range)	6.4 ± 5.1 (-15 to 20)
Coronal deformity ≤ 5°, *n*/*N* (%)	70/209 (33.5%)
Coronal deformity > 5°, *n*/*N* (%)	139/209 (66.5%)
Flexion deformity, degrees, mean ± SD (range)	8.9 ± 7.3 (−13 to 35)
Implant variables	
Implant variables available, *n*	209
Femoral component size #1, *n*/*N* (%)	2/209 (1.0%)
Femoral component size #2, *n*/*N* (%)	36/209 (17.2%)
Femoral component size #3, *n*/*N* (%)	73/209 (34.9%)
Femoral component size #4, *n*/*N* (%)	55/209 (26.3%)
Femoral component size #5, *n*/*N* (%)	34/209 (16.3%)
Femoral component size #6, *n*/*N* (%)	9/209 (4.3%)
Tibial component size #1, *n*/*N* (%)	9/209 (4.3%)
Tibial component size #2, *n*/*N* (%)	40/209 (19.1%)
Tibial component size #3, *n*/*N* (%)	70/209 (33.5%)
Tibial component size #4, *n*/*N* (%)	45/209 (21.5%)
Tibial component size #5, *n*/*N* (%)	31/209 (14.8%)
Tibial component size #6, *n*/*N* (%)	13/209 (6.2%)
Tibial component size #7, *n*/*N* (%)	1/209 (0.5%)
Insert thickness 9 mm, *n*/*N* (%)	192/209 (91.9%)
Insert thickness 11 mm, *n*/*N* (%)	17/209 (8.1%)
Target-gap distribution	
Target gap 17 mm, *n*/*N* (%)	11/836 (1.3%)
Target gap 17.5 mm, *n*/*N* (%)	2/836 (0.2%)
Target gap 18 mm, *n*/*N* (%)	819/836 (98.0%)
Target gap 19 mm, *n*/*N* (%)	4/836 (0.5%)

Values are presented as mean ± standard deviation (range) or *n*/*N* (%), as appropriate. The unit of analysis is the knee unless otherwise specified; 209 knees from 190 patients were included. Percentages were calculated using available data. Percentages may not sum to 100% because of rounding. ASA physical status, implant component sizes, and insert thickness were available for all 209 knees after completion of missing records. Diagnosis was summarized from the completed diagnosis field after trimming whitespace. The “other diagnosis” category comprised hemophilic arthropathy (*n* = 8), Kashin–Beck disease (*n* = 6), and osteonecrosis of the knee (*n* = 5). Target gaps were summarized across 836 gap-level observations. Preoperative coronal deformity was recorded as a signed value, with positive values indicating varus alignment and negative values indicating valgus alignment. ASA, American Society of Anesthesiologists; BMI, body mass index; SD, standard deviation

Relative to the same predefined targets, the model-generated plans showed lower absolute deviation than the preadjustment plans, with MAE decreasing from 2.002 to 1.016 mm (paired Wilcoxon, *p* < 0.001). The proportion of gap-level observations within ±1 mm increased from 49.4% to 77.6%, while the proportion of knees with all four gaps within ±1 mm increased from 11.0% to 43.1%.

### Target-gap concordance

**Fig. 2:**
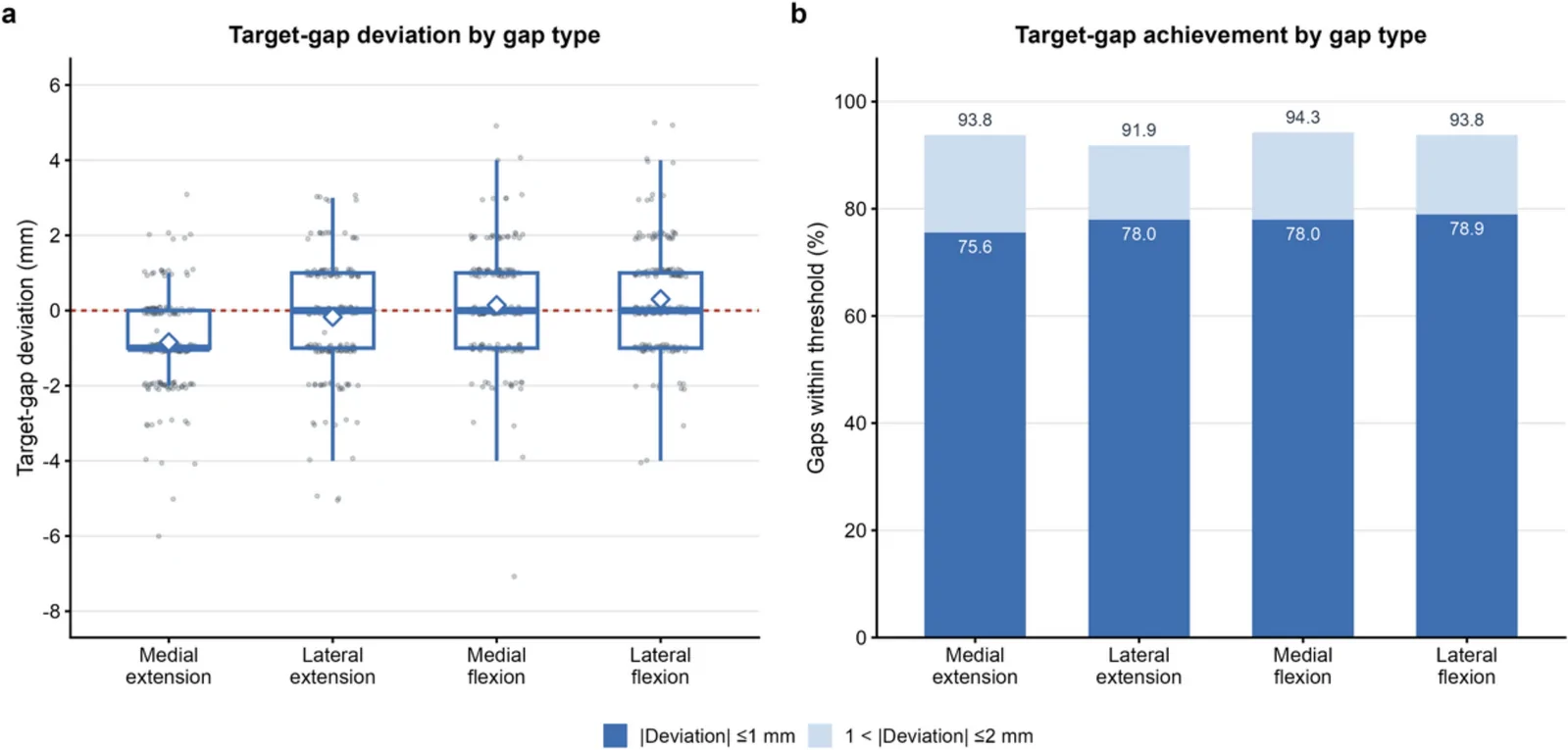
Target-gap deviation and threshold-based achievement by gap type. **a** Distribution of target-gap deviations (software-derived gap minus predefined target gap) by compartment; the red dashed line indicates zero deviation from the predefined target. **b** Stacked proportions of software-derived gaps according to target-gap deviation thresholds. Dark blue indicates gaps within ±1 mm of target, whereas light blue indicates the additional proportion with an absolute deviation > 1 mm and ≤ 2 mm. Accordingly, the total bar height represents the proportion of gaps within ±2 mm of target. ME, medial extension; LE, lateral extension; MF, medial flexion; LF, lateral flexion

Across 836 gap-level observations, the model-generated plans showed a mean signed target-gap deviation of −0.142 mm and an MAE of 1.016 mm. Signed deviations varied modestly across gap types, with the greatest negative bias in medial extension (−0.844 mm) and a small positive bias in lateral flexion (+0.301 mm), whereas absolute deviations were broadly similar across compartments, with MAE ranging from 0.957 to 1.103 mm. One-sample tests identified significant signed deviations in medial extension and lateral flexion, but not in lateral extension or medial flexion. Overall and compartment-specific results are summarized in Table 2, with deviation distributions shown in Fig. 2a and Supplementary Fig. S2.

**Table 2:** Overall and compartment-specific target-gap deviation metrics

Gap type	*n*	Mean signed deviation (mm)	SD (mm)	MAE (mm)	RMSE (mm)	Median AE (mm)	Exact agreement, *n* (%)	Within ±1.0 mm, *n* (%) [95% CI]	Within ±2.0 mm, *n* (%) [95% CI]
Overall (all gap types)	836	−0.142	1.411	1.016	1.417	1.0	262 (31.3)	649 (77.6) [74.7–80.3%]	781 (93.4) [91.5–94.9%]
Medial extension	209	−0.844	1.183	1.103	1.451	1.0	50 (23.9)	158 (75.6) [69.3–80.9%]	196 (93.8) [89.7–96.3%]
Lateral extension	209	− 0.170	1.426	1.031	1.433	1.0	64 (30.6)	163 (78.0) [71.9–83.1%]	192 (91.9) [87.4–94.9%]
Medial flexion	209	0.144	1.393	0.957	1.397	1.0	76 (36.4)	163 (78.0) [71.9–83.1%]	197 (94.3) [90.2–96.7%]
Lateral flexion	209	0.301	1.355	0.971	1.385	1.0	72 (34.4)	165 (78.9) [72.9–83.95]	196 (93.8) [89.7–96.35]

Target-gap deviation was defined as the software-derived gap resulting from the model-generated plan minus the predefined target gap. Negative values indicate tighter-than-target software-derived gaps, whereas positive values indicate larger-than-target gaps. Exact agreement was defined as a 0-mm deviation from the predefined target gap. Threshold achievement indicates the proportion of gap observations within the specified absolute deviation from target. Confidence intervals for the ±1.0-mm and ± 2.0-mm proportions were calculated using Wilson methods. AE, absolute error; MAE, mean absolute error; RMSE, root mean square error; SD, standard deviation. All rates in this table are based on 836 gap-level observations from 209 knees

Overall, 77.6% of software-derived gaps were within ±1.0 mm of target and 93.4% were within ±2.0 mm; exact agreement was observed in 31.3%. Within- ±1.0-mm achievement was similar across the four gap types, ranging from 75.6% to 78.9% (Fig. 2b). Bland–Altman analysis showed a bias of −0.142 mm, with 95% limits of agreement from −2.907 to +2.623 mm; detailed agreement results are provided in Supplementary Table S2 and Supplementary Figs. S1 and S3.

### Knee-level consistency and outliers

At the knee level, the mean per-knee MAE was 1.016 mm (SD, 0.669 mm). Overall, 43.1% of knees had all four software-derived gaps within ±1.0 mm of target, increasing to 83.7% at the ±2.0-mm threshold. In addition, 76.6% of knees had at least three of four gaps within ±1.0 mm, and 92.8% had at least two gaps within this range.

Gap-level outliers, defined as absolute target-gap deviations > 2 mm, occurred in 6.6% of observations, corresponding to 34 knees (16.3%) with at least one outlying gap. Deviations ranged from −7 to +5 mm. Knee-level consistency and outlier metrics are summarized in Table 3; deidentified outlier cases are provided in Supplementary Table S5, and the distribution of per-knee MAE is shown in Supplementary Fig. S4.

**Table 3:** Knee-level consistency and outlier analysis

Metric	Value
Knees analyzed, *n*	209
Per-knee MAE, mm, mean ± SD	1.016 ± 0.669
Per-knee MAE, mm, median [IQR]	1.00 [0.50–1.25]
Per-knee maximum AE, mm, mean ± SD	1.835 ± 1.009
All four gaps within ±1.0 mm, *n/N* (%) [95% CI]	90/209 (43.1%) [36.5–49.8%]
All four gaps within ±2.0 mm, *n/N* (%) [95% CI]	175/209 (83.7%) [78.1–88.1%]
≥ 3 of 4 gaps within ±1.0 mm, *n/N* (%)	160/209 (76.6%)
≥ 2 of 4 gaps within ±1.0 mm, *n/N* (%)	194/209 (92.8%)
Gap-level outliers > 2 mm, *n/N* (%)	55/836 (6.6%)
Knees with ≥ 1 outlier gap, *n/N* (%)	34/209 (16.3%)
Knees with 1 outlier gap, *n*	19
Knees with 2 outlier gaps, *n*	11
Knees with 3 outlier gaps, *n*	2
Knees with 4 outlier gaps, *n*	2
Outliers by compartment: medial extension, *n*	13
Outliers by compartment: lateral extension, *n*	17
Outliers by compartment: medial flexion, *n*	12
Outliers by compartment: lateral flexion, *n*	13
Largest negative target-gap deviation, mm	−7
Largest positive target-gap deviation, mm	+5

Knee-level MAE was calculated as the mean absolute target-gap deviation across the four compartments within each knee. Whole-knee threshold achievement required all four compartments to fall within the specified absolute deviation from target. Gap-level outliers were defined as absolute target-gap deviations > 2 mm. The denominator for knee-level analyses was 209 knees; the denominator for gap-level analyses was 836 observations. AE, absolute error; CI, confidence interval; IQR, interquartile range; MAE, mean absolute error

### Mixed-effects, subgroup, and feasibility analyses

Mixed-effects analysis showed significant differences in signed target-gap deviation across gap types (*p* < 0.001), whereas absolute target-gap deviation did not differ significantly across compartments (*p* = 0.180). The intraclass correlation coefficient from the absolute-error model was 0.277. In exploratory subgroup analyses, male patients had a higher MAE than female patients (1.319 versus 0.888 mm) and lower within- ±1.0-mm target-gap achievement (67.3% versus 82.0%). Performance was similar between knees with ≤ 5° and > 5° of preoperative coronal deformity. Detailed mixed-effects and subgroup results are presented in Table 4 and Supplementary Tables S3 and S4.

**Table 4:** Mixed-effects model results and exploratory subgroup analyses

Panel A. Mixed-effects model summary					
Analysis	Term	Estimate	SE	*p* value	Comment
Signed target-gap deviation	Reference: medial extension (intercept)	−0.845	0.093	< 0.001	Overall gap-type effect *p* < 0.001
Signed target-gap deviation	Lateral extension vs medial extension	0.675	0.120	< 0.001	
Signed target-gap deviation	Medial flexion vs medial extension	0.988	0.120	< 0.001	
Signed target-gap deviation	Lateral flexion vs medial extension	1.146	0.120	< 0.001	
Absolute target-gap deviation	Reference: medial extension (intercept)	1.103	0.068	< 0.001	Overall gap-type effect *p* = 0.180
Absolute target-gap deviation	Lateral extension vs medial extension	−0.072	0.082	0.383	
Absolute target-gap deviation	Medial flexion versus medial extension	−0.146	0.082	0.076	
Absolute target-gap deviation	Lateral flexion versus medial extension	−0.132	0.082	0.110	
Random effects	ICC from absolute-error model	0.277	–	–	Moderate within-knee clustering

Panel B. Exploratory subgroup analyses							
Subgroup	Group	*n*	Bias (mm)	SD (mm)	MAE (mm)	Within ±1.0 mm (%)	Within ±2.0 mm (%)
Target gap	Target ≠ 18 mm	17	−0.176	1.224	0.882	82.4	94.1
Target gap	Target = 18 mm	819	−0.142	1.415	1.018	77.5	93.4
Sex	Female	588	−0.146	1.219	0.888	82.0	95.9
Sex	Male	248	−0.133	1.787	1.319	67.3	87.5
Preoperative coronal deformity magnitude	≤ 5° coronal deformity	280	−0.218	1.416	0.982	78.2	93.2
Preoperative coronal deformity magnitude	> 5° coronal deformity	556	−0.104	1.408	1.032	77.3	93.5

Linear mixed-effects models included gap type as a fixed effect and knee as a random intercept. Medial extension was the reference gap type. The ICC was calculated from the absolute-error model. Subgroup analyses were exploratory and were not adjusted for potential confounders. Subgroup *n* values are reported as gap-level observations. CI, confidence interval; ICC, intraclass correlation coefficient; MAE, mean absolute error

The resection-depth feasibility screen identified one knee (0.5%) with an individual bony resection exceeding the prespecified 10-mm flagging threshold; the lateral tibial resection was 11.5 mm. Five plans contained negative resection values. No other model-generated plan exceeded the 10-mm threshold.

### Secondary plan-reference analysis

Among 206 knees with an available surgeon-approved final plan, model-generated gaps were generally close to the final software gaps, with a mean absolute model-to-final difference of 0.858 mm. Overall, 81.9% and 96.0% were within ±1 and ±2 mm, respectively. Among model-generated outliers, 45 of 55 (81.8%) were corrected to within ±2 mm in the final plan (Supplementary Tables S6–S8 and Supplementary Fig. S5).

## Discussion

This study assessed the software-based concordance between model-generated plans and predefined target gaps within a retrospective robotic planning feedback workflow. The model-generated plans showed a mean absolute deviation of approximately 1 mm, with most software-derived gaps falling within the prespecified tolerance ranges. Interpretation of these findings is nevertheless constrained by the near-uniform target distribution and by the use of software-derived rather than measured intraoperative or postoperative gaps. Thus, the present findings support the model as a surgeon-supervised planning aid within the robotic software environment but do not establish clinical effectiveness. Recent work on predictive gap balancing, ligament tension behavior, spacer- or tensioner-assisted planning, and robotic execution accuracy supports evaluation of planning algorithms at the level of target gap achievement within the planning-feedback loop [17–20].

Although gap deviations may influence knee kinematics and soft-tissue balance, the clinical relevance of the deviations observed in the present study remains uncertain. Because no clinically validated threshold for software-derived gap deviation has been established, the ±1.0-mm tolerance should be interpreted as a pragmatic performance benchmark rather than a clinical cutoff. Nevertheless, evaluating the gap consequences of the complete model-generated plan may be more informative for robotic planning than comparing individual predicted parameters with surgeon-selected values [11–13]. Resection depth, component orientation, component sizing, and their interactions collectively determine the resulting extension and flexion gap profile. Consequently, a numerically small difference in a single predicted parameter may not necessarily translate into a meaningful gap difference, whereas multiple individually modest parameter differences may combine to produce a larger gap deviation. Assessing the plan after its parameters are processed through the robotic planning software therefore provides a workflow-level measure of whether the combined model output converges toward the intended gap profile.

Whole-knee consistency provides a complementary perspective to gap-level performance. Requiring all four extension and flexion gaps to simultaneously meet the same tolerance is inherently more stringent than evaluating each gap independently. A plan may therefore demonstrate favorable overall gap-level performance while still containing an isolated compartment that requires surgeon adjustment. The lower proportion of knees meeting the ±1.0-mm threshold across all four gaps should consequently not be interpreted as inconsistent with the overall gap-level findings, but rather as a more demanding assessment of plan-level concordance. At the same time, most knees had at least three of four gaps within ±1.0 mm, suggesting that the model frequently generated a plan that was already close to the predefined four-gap profile before surgeon refinement. This distinction is potentially important for clinical workflow because a model does not necessarily need to produce an immediately acceptable final plan to be useful; reducing the magnitude or number of subsequent adjustments may itself represent meaningful planning support. Whether such an effect translates into fewer planning iterations or more efficient intraoperative decision-making requires prospective evaluation.

The outlier and subgroup findings further emphasize the importance of maintaining surgeon oversight. A minority of knees contained deviations > 2 mm, indicating that model performance was not uniformly consistent across all cases. Such deviations may reflect patient-specific anatomical or soft-tissue characteristics that are incompletely represented by the model inputs or training data, although the present study was not designed to determine their mechanisms. The exploratory subgroup analyses also showed higher MAE and lower within- ±1.0-mm target-gap achievement in male patients than in female patients, whereas performance was similar between the predefined coronal-deformity strata. These findings should be interpreted cautiously because the subgroup analyses were descriptive rather than inferential and severe deformity was excluded. They nevertheless suggest that model performance may not be identical across patient phenotypes and that future external validation should examine potential sources of performance heterogeneity, including sex, morphology, implant sizing, and deformity severity. More broadly, robotic platforms increasingly support individualized alignment strategies [14, 15, 21–23], and predictive planning systems will ultimately need to demonstrate reliable performance across the broader range of anatomical and alignment phenotypes encountered in routine practice.

The potential workflow implications of the present findings remain hypothesis-generating. Within a surgeon-in-the-loop framework, the model-generated plan may function most appropriately as an initial proposal that narrows the range of subsequent manual adjustments rather than as an autonomous final plan. This interpretation is supported by the secondary plan-reference analysis, in which model-generated gaps were generally close to the surgeon-approved final software gaps and most model-generated outliers were corrected in the final plan. The finding is compatible with a workflow in which the algorithm provides an initial plan that is then reviewed and selectively modified by the surgeon according to the complete intraoperative context. However, the present study did not directly measure the number of planning iterations, time required for plan adjustment, surgeon cognitive workload, or inter-surgeon variability. These outcomes should be incorporated into future prospective evaluations because algorithmic usefulness in the operating room depends not only on numerical concordance but also on whether the technology meaningfully improves decision efficiency without compromising surgeon control. Moreover, both the model-generated and surgeon-approved final gaps in this analysis were software-derived values; therefore, their similarity should be regarded as supportive workflow evidence rather than clinical validation. More broadly, improvements in planning or radiographic precision should not be assumed to translate automatically into superior functional outcomes or implant survivorship [24–29].

This study has several limitations. First, the retrospective single-center design limits generalizability. Although the training and analytic cohorts did not overlap at the case level, they shared the same institution and surgeons; therefore, the present findings represent internal concordance testing and require external validation across different centers, surgeons, and patient populations. Second, 98.0% of target gaps were 18 mm, limiting assessment across heterogeneous and individualized target-gap strategies. Performance across a wider distribution of predefined targets therefore remains uncertain. Third, the predefined targets served both as model inputs and as the reference for concordance assessment, which may inflate apparent achievement. Because a target-withheld analysis was not feasible for the current proprietary implementation, the preadjustment and surgeon-approved final-plan comparisons should be regarded only as contextual benchmarks. Fourth, all study outcomes were derived from the robotic planning software. Measured postresection gaps, trial-component gaps, postoperative gap behavior, clinical outcomes, and workflow endpoints were not assessed, and agreement within the planning environment cannot be assumed to represent equivalent intraoperative or clinical performance. Fifth, the model was developed and evaluated using a single robotic platform and implant system, and its performance cannot be extrapolated to other systems without validation. Finally, severe deformity was excluded, the feasibility assessment was limited to resection depth rather than a comprehensive assessment of alignment and joint-line consequences, and the subgroup analyses were exploratory and descriptive and were not intended for formal between-group inference.

## Conclusions

In this retrospective software-based concordance study, a machine-learning-based planning model generated surgical plans whose robotic software-derived gaps approximated predefined target gaps with a mean absolute deviation of approximately 1 mm. However, compartment-specific bias, moderate whole-knee consistency, and occasional outlier deviations indicate that the model should be used as a surgeon-supervised decision support tool rather than as an autonomous planner. Prospective validation against measured postresection and trial-component gaps, together with clinical and workflow outcomes, is required before clinical effectiveness can be established.

## Supplementary Information

Supplementary Material 1.

Supplementary Material 2.

## Data Availability

No datasets were generated or analyzed during the current study.

## Abbreviations

TKA: Total knee arthroplasty
raTKA: Robotic-assisted total knee arthroplasty
MAE: Mean absolute error
RMSE: Root mean square error
ICC: Intraclass correlation coefficient
LoA: Limits of agreement
ME: Medial extension
LE: Lateral extension
MF: Medial flexion
LF: Lateral flexion
MLP: Multi-layer perceptron
