# Supplementary material for: Software-based concordance analysis of a predictive surgical planning model for target gap achievement in robotic-assisted total knee arthroplasty
Source: Knee Surg Relat Res. 2026 Nov 5;38:56. doi: 10.1186/s43019-026-00357-x (PMC13741710; doi:10.1186/s43019-026-00357-x)
Supplement: Supplementary file 1 [file 43019_2026_357_MOESM1_ESM.docx]

**Supplementary Figure S1. Overall Bland-Altman plot for all 836 gap-level observations.** The mean bias was −0.142 mm, with 95% limits of agreement from −2.907 to +2.623 mm. Points are colored by gap compartment. Target-gap deviation was defined as the software-derived gap resulting from the model-generated plan minus the predefined target gap. Because most target gaps were 18 mm, this plot should be interpreted as a summary of deviation from target rather than as evidence of agreement across a wide range of target values.

**Supplementary Figure S2. Histograms of target-gap deviations by gap type.** Histograms showing the distribution of target-gap deviations for medial extension, lateral extension, medial flexion, and lateral flexion. The dashed vertical line indicates zero deviation from target, and the solid red line indicates the mean bias for each compartment. Negative values indicate tighter-than-target software-derived gaps, whereas positive values indicate larger-than-target gaps.

**Supplementary Figure S3. Model-plan-derived software gaps versus predefined target gaps by gap type.** Scatter plots comparing software-derived gaps resulting from model-generated plans with predefined target gaps for each compartment. The dashed line represents the identity line, and dotted lines indicate ±1 mm bounds around the target. Because target gaps were predominantly 18 mm, these plots primarily show vertical dispersion of software-derived gaps around the target rather than correlation across a broad target range.

S**upplementary Figure S4. Per-knee mean absolute target-gap deviation.** Waterfall plot showing per-knee mean absolute error across the four gap compartments, sorted in ascending order. Each bar represents one knee. Bar color indicates whole-knee threshold classification: all four gaps within ±1 mm, all four gaps within ±2 mm but not all within ±1 mm, or at least one gap outside ±2 mm. The dashed horizontal line indicates 1 mm mean absolute error.

**Supplementary Figure S5. Secondary plan-reference analysis comparing model-generated software gaps with surgeon-approved final software gaps.** Panel A shows the distribution of model-to-final differences by gap type, defined as model-generated software-derived gap minus surgeon-approved final software-derived gap. Reference lines indicate 0 mm and ±1 mm and ±2 mm differences. Panel B shows Bland-Altman plots comparing model-generated software gaps with surgeon-approved final software gaps by gap type; the solid red line indicates the mean bias and dashed red lines indicate the 95% limits of agreement. Panel C compares absolute target-gap deviation between the model-generated plan and the surgeon-approved final plan, with paired lines linking observations from the same gap compartment within the same knee. Panel D shows the correction status of model-generated plan outliers, defined as absolute target-gap deviation >2 mm, after surgeon-approved final plan adjustment. Surgeon-approved final gaps were software-derived gaps from the final robotic plan after surgeon adjustment and approval and should not be interpreted as trial-component measured gaps, actual intraoperative gap measurements, or postoperative clinical outcomes.
