# Supplementary material for: Software-based concordance analysis of a predictive surgical planning model for target gap achievement in robotic-assisted total knee arthroplasty
Source: Knee Surg Relat Res. 2026 Nov 5;38:56. doi: 10.1186/s43019-026-00357-x (PMC13741710; doi:10.1186/s43019-026-00357-x)
Supplement: Supplementary file 2 [file 43019_2026_357_MOESM2_ESM.docx]

**Supplementary Table S1. Variables used by the predictive planning model and in the planning-feedback concordance workflow.**

***Panel A. Inference-time input and target/reference variables***

| **Category** | **Variable** | **Role in the current workflow** | **Unit** | **Source / measurement method** |
| --- | --- | --- | --- | --- |
| Pre-adjustment planning variable | Medial distal femoral resection | Input available before final plan adjustment | mm | Initial robotic plan before final adjustment |
| Pre-adjustment planning variable | Lateral distal femoral resection | Input available before final plan adjustment | mm | Initial robotic plan before final adjustment |
| Pre-adjustment planning variable | Femoral varus/valgus angle | Input available before final plan adjustment | ° | Initial robotic plan before final adjustment |
| Pre-adjustment planning variable | Medial posterior condylar femoral resection | Input available before final plan adjustment | mm | Initial robotic plan before final adjustment |
| Pre-adjustment planning variable | Lateral posterior condylar femoral resection | Input available before final plan adjustment | mm | Initial robotic plan before final adjustment |
| Pre-adjustment planning variable | Femoral internal/external rotation angle | Input available before final plan adjustment | ° | Initial robotic plan before final adjustment |
| Pre-adjustment planning variable | Medial proximal tibial resection | Input available before final plan adjustment | mm | Initial robotic plan before final adjustment |
| Pre-adjustment planning variable | Lateral proximal tibial resection | Input available before final plan adjustment | mm | Initial robotic plan before final adjustment |
| Pre-adjustment planning variable | Tibial varus/valgus angle | Input available before final plan adjustment | ° | Initial robotic plan before final adjustment |
| Pre-adjustment gap measurement | Medial extension gap | Input available before final plan adjustment | mm | Intraoperative robotic measurement before final plan adjustment |
| Pre-adjustment gap measurement | Lateral extension gap | Input available before final plan adjustment | mm | Intraoperative robotic measurement before final plan adjustment |
| Pre-adjustment gap measurement | Medial flexion gap | Input available before final plan adjustment | mm | Intraoperative robotic measurement before final plan adjustment |
| Pre-adjustment gap measurement | Lateral flexion gap | Input available before final plan adjustment | mm | Intraoperative robotic measurement before final plan adjustment |
| Predefined target-gap variable | Target medial extension gap | Target/reference variable for the concordance analysis | mm | Predefined within the robotic planning workflow; not derived from the surgeon-approved final plan |
| Predefined target-gap variable | Target lateral extension gap | Target/reference variable for the concordance analysis | mm | Predefined within the robotic planning workflow; not derived from the surgeon-approved final plan |
| Predefined target-gap variable | Target medial flexion gap | Target/reference variable for the concordance analysis | mm | Predefined within the robotic planning workflow; not derived from the surgeon-approved final plan |
| Predefined target-gap variable | Target lateral flexion gap | Target/reference variable for the concordance analysis | mm | Predefined within the robotic planning workflow; not derived from the surgeon-approved final plan |

***Panel B. Model-generated outputs and software-derived gaps***

| **Category** | **Variable** | **Role in the current workflow** | **Unit** | **Source / measurement method** |
| --- | --- | --- | --- | --- |
| Model-generated planning output | Model-generated femoral varus/valgus angle | Model output used to construct the model-generated plan | ° | Predictive planning model output; returned to the robotic planning software |
| Model-generated planning output | Model-generated medial distal femoral resection | Model output used to construct the model-generated plan | mm | Predictive planning model output; returned to the robotic planning software |
| Model-generated planning output | Model-generated lateral distal femoral resection | Model output used to construct the model-generated plan | mm | Predictive planning model output; returned to the robotic planning software |
| Model-generated planning output | Model-generated femoral rotation based on posterior condylar axis | Model output used to construct the model-generated plan | ° | Predictive planning model output; returned to the robotic planning software |
| Model-generated planning output | Model-generated femoral rotation based on transepicondylar axis | Model output used to construct the model-generated plan | ° | Predictive planning model output; returned to the robotic planning software |
| Model-generated planning output | Model-generated medial posterior condylar femoral resection | Model output used to construct the model-generated plan | mm | Predictive planning model output; returned to the robotic planning software |
| Model-generated planning output | Model-generated lateral posterior condylar femoral resection | Model output used to construct the model-generated plan | mm | Predictive planning model output; returned to the robotic planning software |
| Model-generated planning output | Model-generated femoral rollback | Model output used to construct the model-generated plan | mm | Predictive planning model output; returned to the robotic planning software |
| Model-generated planning output | Model-generated tibial varus/valgus angle | Model output used to construct the model-generated plan | ° | Predictive planning model output; returned to the robotic planning software |
| Model-generated planning output | Model-generated medial proximal tibial resection | Model output used to construct the model-generated plan | mm | Predictive planning model output; returned to the robotic planning software |
| Model-generated planning output | Model-generated lateral proximal tibial resection | Model output used to construct the model-generated plan | mm | Predictive planning model output; returned to the robotic planning software |
| Model-generated planning output | Model-generated tibial rotation | Model output used to construct the model-generated plan | ° | Predictive planning model output; returned to the robotic planning software |
| Model-generated planning output | Model-generated tibial slope | Model output used to construct the model-generated plan | ° | Predictive planning model output; returned to the robotic planning software |
| Software-derived gap | Software-derived medial extension gap | Primary outcome component | mm | Calculated by the robotic planning software after the model-generated plan was imported |
| Software-derived gap | Software-derived lateral extension gap | Primary outcome component | mm | Calculated by the robotic planning software after the model-generated plan was imported |
| Software-derived gap | Software-derived medial flexion gap | Primary outcome component | mm | Calculated by the robotic planning software after the model-generated plan was imported |
| Software-derived gap | Software-derived lateral flexion gap | Primary outcome component | mm | Calculated by the robotic planning software after the model-generated plan was imported |

**Note.** Variables are grouped according to their role in the current planning-feedback concordance workflow. Pre-adjustment variables were available before final plan adjustment. Predefined target-gap variables were not derived from the surgeon-approved final plan. Model-generated planning outputs were returned to the robotic planning software, which calculated the software-derived gaps used for target-gap deviation analysis.

**Supplementary Table S2. Detailed Bland-Altman and one-sample bias statistics.**

***Panel A. Detailed Bland-Altman statistics overall and by gap type***

| **Gap type** | **n** | **Bias, mm** | **95% CI for bias, mm** | **SD, mm** | **Lower LoA, mm** | **Upper LoA, mm** |
| --- | --- | --- | --- | --- | --- | --- |
| Overall | 836 | -0.142 | -0.238 to -0.047 | 1.411 | -2.907 | 2.623 |
| Medial Extension | 209 | -0.844 | -1.005 to -0.684 | 1.183 | -3.164 | 1.475 |
| Lateral Extension | 209 | -0.170 | -0.363 to 0.024 | 1.426 | -2.966 | 2.626 |
| Medial Flexion | 209 | 0.144 | -0.045 to 0.332 | 1.393 | -2.587 | 2.874 |
| Lateral Flexion | 209 | 0.301 | 0.118 to 0.485 | 1.355 | -2.355 | 2.958 |

***Panel B. One-sample bias tests by gap type***

| **Gap type** | **n** | **Mean bias, mm** | **SD, mm** | **SE, mm** | **t statistic** | **p value** | **95% CI, mm** |
| --- | --- | --- | --- | --- | --- | --- | --- |
| Medial Extension | 209 | -0.845 | 1.183 | 0.082 | -10.318 | <0.001 | -1.006 to -0.683 |
| Lateral Extension | 209 | -0.170 | 1.426 | 0.099 | -1.722 | 0.087 | -0.364 to 0.025 |
| Medial Flexion | 209 | 0.143 | 1.393 | 0.096 | 1.490 | 0.138 | -0.046 to 0.334 |
| Lateral Flexion | 209 | 0.301 | 1.355 | 0.094 | 3.216 | 0.002 | 0.117 to 0.486 |

**Note.** Bias was calculated as the mean target-gap deviation, defined as the software-derived gap resulting from the model-generated plan minus the predefined target gap. Limits of agreement were calculated as mean bias ± 1.96 × SD. One-sample t-tests assessed whether the mean signed target-gap deviation differed from zero. LoA, limits of agreement; SD, standard deviation; SE, standard error.

**Supplementary Table S3. Full mixed-effects model outputs.**

| **Model** | **Effect** | **Term** | **Estimate** | **SE** | **Statistic** | **df** | **p value** |
| --- | --- | --- | --- | --- | --- | --- | --- |
| Signed target-gap deviation | Fixed | Intercept (medial extension reference) | -0.845 | 0.093 | -9.092 | 765.9 | <0.001 |
| Signed target-gap deviation | Fixed | Lateral extension vs medial extension | 0.675 | 0.120 | 5.636 | 624.0 | <0.001 |
| Signed target-gap deviation | Fixed | Medial flexion vs medial extension | 0.988 | 0.120 | 8.254 | 624.0 | <0.001 |
| Signed target-gap deviation | Fixed | Lateral flexion vs medial extension | 1.146 | 0.120 | 9.573 | 624.0 | <0.001 |
| Absolute target-gap deviation | Fixed | Intercept (medial extension reference) | 1.103 | 0.068 | 16.124 | 676.1 | <0.001 |
| Absolute target-gap deviation | Fixed | Lateral extension vs medial extension | -0.072 | 0.082 | -0.873 | 624.0 | 0.383 |
| Absolute target-gap deviation | Fixed | Medial flexion vs medial extension | -0.146 | 0.082 | -1.775 | 624.0 | 0.076 |
| Absolute target-gap deviation | Fixed | Lateral flexion vs medial extension | -0.132 | 0.082 | -1.600 | 624.0 | 0.110 |
| Within ±1.0 mm target-gap achievement (logit) | Fixed | Intercept (medial extension reference) | 1.323 | 0.193 | 6.864 |  | <0.001 |
| Within ±1.0 mm target-gap achievement (logit) | Fixed | Lateral extension vs medial extension | 0.152 | 0.247 | 0.617 |  | 0.537 |
| Within ±1.0 mm target-gap achievement (logit) | Fixed | Medial flexion vs medial extension | 0.152 | 0.247 | 0.617 |  | 0.537 |
| Within ±1.0 mm target-gap achievement (logit) | Fixed | Lateral flexion vs medial extension | 0.216 | 0.249 | 0.869 |  | 0.385 |

**Note.** Mixed-effects models included gap type as a fixed categorical effect and knee as a random intercept. Medial extension was the reference gap type. The signed-deviation model showed a significant overall gap-type effect (p < 0.001). The absolute-deviation model showed no significant overall gap-type effect (p = 0.180). The intraclass correlation coefficient from the absolute-error mixed-effects model was 0.277, indicating moderate within-knee clustering of absolute error. SE, standard error.

**Supplementary Table S4. Exploratory subgroup analyses.**

| **Subgroup** | **Group** | **n** | **Mean bias, mm** | **SD, mm** | **MAE, mm** | **Within** ±**1.0 mm, %** | **Within** ±**2.0 mm, %** |
| --- | --- | --- | --- | --- | --- | --- | --- |
| Target-gap category | Target ≠ 18 mm | 17 | -0.176 | 1.224 | 0.882 | 82.4 | 94.1 |
| Target-gap category | Target = 18 mm | 819 | -0.142 | 1.415 | 1.018 | 77.5 | 93.4 |
| Sex | Female | 588 | -0.146 | 1.219 | 0.888 | 82.0 | 95.9 |
| Sex | Male | 248 | -0.133 | 1.787 | 1.319 | 67.3 | 87.5 |
| Preoperative coronal deformity magnitude | ≤5° coronal deformity | 280 | -0.218 | 1.416 | 0.982 | 78.2 | 93.2 |
| Preoperative coronal deformity magnitude | >5° coronal deformity | 556 | -0.104 | 1.408 | 1.032 | 77.3 | 93.5 |

**Note.** Subgroup analyses were exploratory and were not adjusted for potential confounders. Subgroup sample sizes are reported as gap-level observations. Bias represents mean signed target-gap deviation. MAE, mean absolute error; SD, standard deviation.

**Supplementary Table S5. Deidentified outlier cases with absolute target-gap deviation greater than 2 mm.**

| **Outlier ID** | **Age group, y** | **Sex** | **Preop coronal deformity, deg** | **Preop flexion deformity, deg** | **Femoral size** | **Tibial size** | **Insert, mm** | **ME dev., mm** | **LE dev., mm** | **MF dev., mm** | **LF dev., mm** | **Max AE, mm** | **Knee MAE, mm** |
| --- | --- | --- | --- | --- | --- | --- | --- | --- | --- | --- | --- | --- | --- |
| O01 | <50 | Male | 15 | 20 | 4 | 5 | 9 | -6 | -5 | -7 | 1 | 7 | 4.75 |
| O02 | <50 | Male | 5 | 8 | 3 | 3 | 9 | -5 | -5 | -4 | -4 | 5 | 4.50 |
| O03 | <50 | Male | -3 | -1 | 3 | 4 | 11 | 1 | -5 | -1 | 0 | 5 | 1.75 |
| O04 | 50-59 | Female | 16 | 10 | 3 | 2 | 9 | -3 | 3 | 3 | 5 | 5 | 3.50 |
| O05 | <50 | Male | -1 | 25 | 3 | 3 | 9 | 0 | -3 | 5 | 5 | 5 | 3.25 |
| O06 | 60-69 | Female | -15 | -6 | 3 | 2 | 9 | 2 | 0 | 4 | -1 | 4 | 1.75 |
| O07 | 60-69 | Female | 6 | 2 | 3 | 3 | 9 | -1 | -1 | -1 | -4 | 4 | 1.75 |
| O08 | Missing | Male | 4 | 19 | 3 | 3 | 9 | -3 | -4 | -1 | 2 | 4 | 2.50 |
| O09 | 60-69 | Female | 14 | 14 | 3 | 3 | 9 | -1 | -1 | -1 | 4 | 4 | 1.75 |
| O10 | 60-69 | Female | 0 | 6 | 3 | 3 | 9 | -1 | -4 | 2 | 3 | 4 | 2.50 |
| O11 | 50-59 | Male | 16 | 27 | 5 | 4 | 11 | -4 | 3 | -2 | 2 | 4 | 2.75 |
| O12 | 60-69 | Male | 10 | 12 | 6 | 7 | 9 | -4 | 1 | 0 | -1 | 4 | 1.50 |
| O13 | 70-79 | Male | 8 | 13 | 4 | 5 | 9 | -1 | -1 | -1 | 4 | 4 | 1.75 |
| O14 | 70-79 | Female | 7 | 6 | 4 | 4 | 9 | 0 | 1 | 3 | 4 | 4 | 2.00 |
| O15 | 60-69 | Female | 1 | 12 | 2 | 2 | 9 | 3 | 1 | 4 | 2 | 4 | 2.50 |
| O16 | 70-79 | Female | 16 | 4 | 2 | 2 | 9 | -4 | -2 | 0 | -1 | 4 | 1.75 |
| O17 | 70-79 | Female | 16 | 13 | 3 | 3 | 9 | -1 | 3 | -2 | 0 | 3 | 1.50 |
| O18 | 60-69 | Male | 12 | 19 | 6 | 6 | 9 | -3 | 2 | -2 | 0 | 3 | 1.75 |
| O19 | 70-79 | Female | 7 | 3 | 3 | 3 | 9 | 0 | 1 | 0 | 3 | 3 | 1.00 |
| O20 | Missing | Male | 12 | 9 | 6 | 5 | 9 | -3 | 3 | 0 | 1 | 3 | 1.75 |
| O21 | 70-79 | Female | 19 | 11 | 4 | 4 | 9 | -2 | 3 | -1 | 1 | 3 | 1.75 |
| O22 | Missing | Male | 10 | 3 | 6 | 6 | 9 | -1 | 1 | -1 | 3 | 3 | 1.50 |
| O23 | Missing | Male | 7 | 13 | 5 | 6 | 9 | -1 | 1 | 0 | 3 | 3 | 1.25 |
| O24 | Missing | Male | 10 | 4 | 5 | 6 | 9 | -3 | -2 | -3 | -1 | 3 | 2.25 |
| O25 | 30-39 | Male | 4 | 6 | 4 | 4 | 9 | -2 | -3 | -3 | 0 | 3 | 2.00 |
| O26 | <50 | Male | 17 | 18 | 4 | 5 | 9 | -3 | 2 | 1 | 0 | 3 | 1.50 |
| O27 | <50 | Female | -2 | 11 | 4 | 4 | 9 | -1 | -3 | -1 | -3 | 3 | 2.00 |
| O28 | 60-69 | Male | 6 | 9 | 5 | 5 | 9 | 2 | 3 | -2 | -2 | 3 | 2.25 |
| O29 | 60-69 | Female | -5 | 7 | 2 | 2 | 11 | 0 | -3 | 0 | -1 | 3 | 1.00 |
| O30 | 70-79 | Female | 8 | 8 | 4 | 3 | 9 | -2 | -3 | -1 | 1 | 3 | 1.75 |
| O31 | 60-69 | Male | 11 | 19 | 4 | 4 | 9 | 0 | 2 | 3 | 2 | 3 | 1.75 |
| O32 | 70-79 | Female | -6 | 16 | 3 | 2 | 9 | 2 | -3 | 1 | 0 | 3 | 1.50 |
| O33 | 70-79 | Female | 8 | 35 | 4 | 4 | 9 | -1 | -1 | 3 | 3 | 3 | 2.00 |
| O34 | <50 | Male | 8 | 20 | 5 | 5 | 11 | -3 | -1 | 3 | 2 | 3 | 2.25 |

**Note.** Outlier cases were defined as knees with at least one compartment showing an absolute target-gap deviation >2 mm. Patient identifiers, case IDs, exact surgery dates, and free-text notes were removed. Age is reported by group to reduce re-identification risk. Deviation was calculated as software-derived gap minus predefined target gap. ME, medial extension; LE, lateral extension; MF, medial flexion; LF, lateral flexion; AE, absolute error; MAE, mean absolute error. Positive coronal deformity values indicate varus alignment, and negative values indicate valgus alignment.

**Supplementary Table S6. Agreement between model-generated software gaps and surgeon-approved final software gaps.**

| Gap type | n | Mean signed model-to-final difference, mm | SD, mm | MAE, mm | RMSE, mm | Median absolute difference, mm | IQR of absolute difference, mm | Within ±1.0 mm, n (%) [95% CI] | Within ±2.0 mm, n (%) [95% CI] | Min signed difference, mm | Max signed difference, mm |
| --- | --- | --- | --- | --- | --- | --- | --- | --- | --- | --- | --- |
| Overall | 824 | 0.047 | 1.322 | 0.858 | 1.322 | 1 | 0.000-1.000 | 675/824 (81.9%) [79.1-84.4%] | 791/824 (96.0%) [94.4-97.1%] | -8 | 9 |
| Medial Extension | 206 | -0.034 | 1.463 | 0.917 | 1.460 | 1 | 0.000-1.000 | 160/206 (77.7%) [71.5-82.8%] | 198/206 (96.1%) [92.5-98.0%] | -7 | 9 |
| Lateral Extension | 206 | 0.209 | 1.284 | 0.917 | 1.298 | 1 | 0.000-1.000 | 166/206 (80.6%) [74.6-85.4%] | 197/206 (95.6%) [91.9-97.7%] | -7 | 3 |
| Medial Flexion | 206 | -0.083 | 1.382 | 0.869 | 1.381 | 1 | 0.000-1.000 | 171/206 (83.0%) [77.3-87.5%] | 198/206 (96.1%) [92.5-98.0%] | -8 | 7 |
| Lateral Flexion | 206 | 0.097 | 1.126 | 0.728 | 1.128 | 1 | 0.000-1.000 | 178/206 (86.4%) [81.1-90.4%] | 198/206 (96.1%) [92.5-98.0%] | -4 | 4 |

**Note.** Model-to-final difference was defined as model-generated software-derived gap minus surgeon-approved final software-derived gap. The final gaps are software-derived gaps from the surgeon-approved robotic plan and should not be interpreted as actual intraoperative or postoperative measured gaps. CI, confidence interval; IQR, interquartile range; MAE, mean absolute error; RMSE, root mean square error; SD, standard deviation.

**Supplementary Table S7. Target-gap achievement before and after surgeon-supervised final plan adjustment.**

| Plan stage | Gap type | n | Mean signed deviation from target, mm | MAE, mm | RMSE, mm | Median AE, mm | Within ±1.0 mm, n (%) | Within ±2.0 mm, n (%) |
| --- | --- | --- | --- | --- | --- | --- | --- | --- |
| Model-generated plan | Overall | 824 | -0.144 | 1.021 | 1.424 | 1 | 637/824 (77.3%) | 769/824 (93.3%) |
| Model-generated plan | Medial Extension | 206 | -0.852 | 1.104 | 1.457 | 1 | 155/206 (75.2%) | 193/206 (93.7%) |
| Model-generated plan | Lateral Extension | 206 | -0.177 | 1.041 | 1.442 | 1 | 160/206 (77.7%) | 189/206 (91.7%) |
| Model-generated plan | Medial Flexion | 206 | 0.150 | 0.956 | 1.402 | 1 | 160/206 (77.7%) | 194/206 (94.2%) |
| Model-generated plan | Lateral Flexion | 206 | 0.301 | 0.981 | 1.393 | 1 | 162/206 (78.6%) | 193/206 (93.7%) |
| Surgeon-approved final plan | Overall | 824 | -0.192 | 0.867 | 1.240 | 1 | 689/824 (83.6%) | 793/824 (96.2%) |
| Surgeon-approved final plan | Medial Extension | 206 | -0.818 | 1.148 | 1.530 | 1 | 148/206 (71.8%) | 192/206 (93.2%) |
| Surgeon-approved final plan | Lateral Extension | 206 | -0.386 | 1.017 | 1.332 | 1 | 162/206 (78.6%) | 195/206 (94.7%) |
| Surgeon-approved final plan | Medial Flexion | 206 | 0.233 | 0.592 | 1.014 | 0 | 193/206 (93.7%) | 204/206 (99.0%) |
| Surgeon-approved final plan | Lateral Flexion | 206 | 0.204 | 0.709 | 1.005 | 1 | 186/206 (90.3%) | 202/206 (98.1%) |

**Note.** Target-gap deviation was defined as software-derived gap minus predefined target gap. The surgeon-approved final plan represents the final software-derived robotic plan accepted by the surgeon and is not an independent clinical gold standard. AE, absolute error; MAE, mean absolute error; RMSE, root mean square error. All rates in this table are based on 824 gap-level observations from the 206 knees with an available surgeon-approved final plan.

**Supplementary Table S8. Outlier correction by the surgeon-approved final software plan.**

***Panel A. Overall gap-level outlier correction***

| Metric | Numerator | Denominator | n/N (%) |
| --- | --- | --- | --- |
| Gap-level model outliers | 55 | 836 | 55/836 (6.6%) |
| Corrected to within ±2 mm | 45 | 55 | 45/55 (81.8%) |
| Corrected to within ±1 mm | 35 | 55 | 35/55 (63.6%) |
| Persistent outliers | 10 | 55 | 10/55 (18.2%) |
| New final-plan outliers | 21 | 769 | 21/769 (2.7%) |

***Panel B. Outlier correction by gap type***

| Gap type | Gap-level observations | Model outliers, n (%) | Corrected to within ±2 mm among model outliers | Corrected to within ±1 mm among model outliers | Persistent outliers among model outliers | New final-plan outliers among non-model outliers |
| --- | --- | --- | --- | --- | --- | --- |
| Medial Extension | 209 | 13/209 (6.2%) | 9/13 (69.2%) | 8/13 (61.5%) | 4/13 (30.8%) | 10/193 (5.2%) |
| Lateral Extension | 209 | 17/209 (8.1%) | 13/17 (76.5%) | 9/17 (52.9%) | 4/17 (23.5%) | 7/189 (3.7%) |
| Medial Flexion | 209 | 12/209 (5.7%) | 11/12 (91.7%) | 8/12 (66.7%) | 1/12 (8.3%) | 1/194 (0.5%) |
| Lateral Flexion | 209 | 13/209 (6.2%) | 12/13 (92.3%) | 10/13 (76.9%) | 1/13 (7.7%) | 3/193 (1.6%) |

***Panel C. Knee-level outlier correction***

| Metric | Numerator | Denominator | n/N (%) |
| --- | --- | --- | --- |
| Knees with at least one model outlier | 34 | 209 | 34/209 (16.3%) |
| Among knees with model outlier: no final-plan outlier | 21 | 34 | 21/34 (61.8%) |
| Among knees with model outlier: at least one persistent/final outlier | 13 | 34 | 13/34 (38.2%) |
| Among knees without model outlier: new final-plan outlier | 14 | 172 | 14/172 (8.1%) |

**Note.** Model-outlier prevalence was calculated in the full analytic cohort of 209 knees and 836 gap-level observations (55/836 gap-level observations and 34/209 knees). Correction analyses were conducted among the 206 knees and 824 gap-level observations with an available surgeon-approved final software plan.
